# Causal effects and metabolites mediators between immune cell and risk of breast cancer: a Mendelian randomization study

**DOI:** 10.3389/fgene.2024.1380249

**Published:** 2024-05-17

**Authors:** Ruijie Ming, Huan Wu, Hong Liu, Fangbiao Zhan, Xingan Qiu, Ming Ji

**Affiliations:** ^1^ Department of Oncology, Chongqing University Three Gorges Hospital, Chongqing, China; ^2^ School of Medicine, University of Electronic Science and Technology of China, Chengdu, China; ^3^ Department of Orthopedics, Chongqing University Three Gorges Hospital, Chongqing, China

**Keywords:** immune cell, Mendelian randomization study, breast cancer, causal effect, metabolites mediator

## Abstract

**Introduction:** The incidence and mortality of female breast cancer remain high, and the immune microenvironment of breast cancer has undergone significant alterations. However, the impact of blood immune cell levels on the risk of breast cancer is not fully understood. Therefor this study aims to investigate the causal relationship between blood immune cell levels and the risk of breast cancer.

**Methods:** A Mendelian randomization (MR) analysis was employed to assess the causal relationship between immune cells and the risk of breast cancer, as along with their potential mediating factors. Genetic statistics of metabolites breast cancer and immune cells were obtained from the GWAS Catalog, while the genome-wide association study (GWAS) statistics of breast cancer were extracted from the UK biobank. Two-sample MR analysis were performed using inverse-variance weighted (IVW) to ascertain the causal association between immune cells and the risk of breast cancer. Furthermore, 1,400 metabolites were analyzed for their mediating role between immune cells and the risk of breast cancer.

**Results:** MR analysis through IVW method revealed that genetically predicted CD24^+^ CD27^+^ B cells were associated with a decreased risk of breast cancer (OR = 0.9978, 95% CI: 0.996–0.999, *p* = 0.001), while IgD- CD38^+^ B cells were linked to an increased risk of breast cancer (OR = 1.002, 95% CI: 1.001–1.004, *p* = 0.005). Additional CD14^+^ CD16^+^ monocytes were associated with an increased risk of breast cancer (OR = 1.000, 95% CI: 1.000–1.001, *p* = 0.005). Mediation analysis revealed a positive causal relationship between IgD- CD38^+^ B cells and Glycerate levels, with the latter also exhibiting a positive causal relationship with the risk of breast cancer (*p* < 0.05). Conversely, IgD- CD38^+^ B cells displayed a negative causal relationship with Succinoyltaurine levels, and the latter also demonstrated a negative causal relationship with the risk of breast cancer (*p* < 0.05).

**Conclusion:** This MR study provides novel genetic evidence supporting a causal relationship between IgD- CD38^+^ B cells and the risk of BC. Moreover, it is identified that IgD- CD38^+^ B cells contribute to an increased risk of BC through both positive and negative mediation effects involving Glycerate and Succinoyltaurine.

## 1 Introduction

Breast cancer (BC) has emerged as the most prevalent malignancy among women worldwide, with a mortality rate that ranks second only to lung cancer. It is estimated that there will be approximately 43,170 deaths (6.6%) in the United States in 2023 (Siegel et al., 2023). From 2008 to 2022, the global incidence of BC experienced a significant increase of 20%, while the mortality rate rose by 14% (Sher et al., 2022). This alarming trend is reflected in the estimated global burden of BC, which surged to 2.26 million new cases in 2020, a substantial increase from the nearly 1.7 million cases recorded in 2012 (Torre et al., 2017; Sung et al., 2021).

A growing body of research highlights the critical role of the tumor microenvironment (TME) in the development and progression of BC (Denkert et al., 2018; Pruneri et al., 2018). The TME is a complex ecosystem composed of tumor cells, stromal cells, immune cells, metabolites, and secreted proteins. Recent advances in TME research have revealed that the remodeling of metabolism within TME also plays a pivotal role in suppressing tumor immunity. For instance, the excessive production of metabolic by-products can disrupt the metabolic rewiring of T cells, thereby impairing their anti-tumor function (Park et al., 2023). Therefore, investigating the immune cells and metabolites that causal influence the risk of BC is of paramount importance. Such exploration is essential for elucidating the mechanisms underlying the immune microenvironment in the progression of BC.

Traditionally, meticulously planned randomized controlled trials (RCTs) have been considered as the gold standard for establishing causal relationships between exposure factors and outcomes. However, their implementation often requires ethical approval and extensive follow-up, making them complex and resource-intensive (Smylie et al., 2024). To efficiently identify potential disease-exposure relationships during the exploratory stage, Mendelian randomization (MR) analysis has emerged as a powerful epidemiological tool. MR is commonly employed to assess the causal relationship between exposure factors and outcomes by utilizing genetic variants, known as single nucleotide polymorphisms (SNPs), as instrumental variables (IVs) (Birney, 2022). These IVs act as effective proxies for observed exposures, providing more robust estimates of causality compared to traditional observational studies (Fang et al., 2024). Recent MR studies have demonstrated that SNPs related to immune cells can serve as exposure factors influencing tumor development (Bouras et al., 2022; Yu et al., 2022; Aru et al., 2023; Yin et al., 2023). However, the relationship between immune cells, metabolites, and BC has not been explored using MR.

In this study, MR was employed to identify immune cells significantly impacting BC incidence. Subsequently, reverse MR was applied to determine that BC did not affect the levels of these immune cells. Next, the SNPs related to metabolites were selected as intermediaries. Finally, through mediation analysis, we discovered that IgD- CD38^+^ B cells could elevate the risk of BC by influencing Succinoyltaurine and Glycerate.

## 2 Materials and methods

### 2.1 Datasets acquisition

The datasets utilized in this study are summarized in Table 1. The summary genome-wide association study (GWAS) statistics of immune cells were obtained from the MRCEU open database (accession numbers ranging from GCST90001391 to GCST90002121) (Elsworth et al., 2020; Orrù et al., 2020). These data encompass 731 immune cells with different trait types (details in Suplementary Table S1), including absolute count (*n* = 118), median fluorescence intensities (MFI, *n* = 389), morphological parameters (*n* = 32), and relative count (*n* = 192). The GWAS statistics for BC were acquired from the UK Biobank, which includes 10,303 BC cases and 452,630 healthy controls, encompassing total 9,851,867 SNPs (Rusk, 2018; Elsworth et al., 2020).

**TABLE 1:** Datasets sources.

Phenotypes	Datasets source	Phenotype code	Cases/Controls	Ancestry
Breast cancer	UK Biobank Rusk (2018)	ukb-b-16890	10303/452630	European
Immune cells	Cucca et al. Orrù et al. (2020)	GCST0001391 to GCST0002121		European
Metabolites	Richards et al. Chen et al. (2023)	GCST90199621 to GCST90201020		European

We accessed metabolites GWAS statistics to investigate its mediating effect. Summary GWAS statistics for metabolites were retrieved from the GWAS Catalog, identified with accession numbers ranging from GCST90199621 to GCST90201020 (Chen et al., 2023). A total of 1,400 metabolites were included in our study, as outlined in Suplementary Table S2. By integrating data from these diverse sources, we aimed to obtain a comprehensive dataset for our research.

### 2.2 Identification of instrumental variables

**FIGURE 1:**
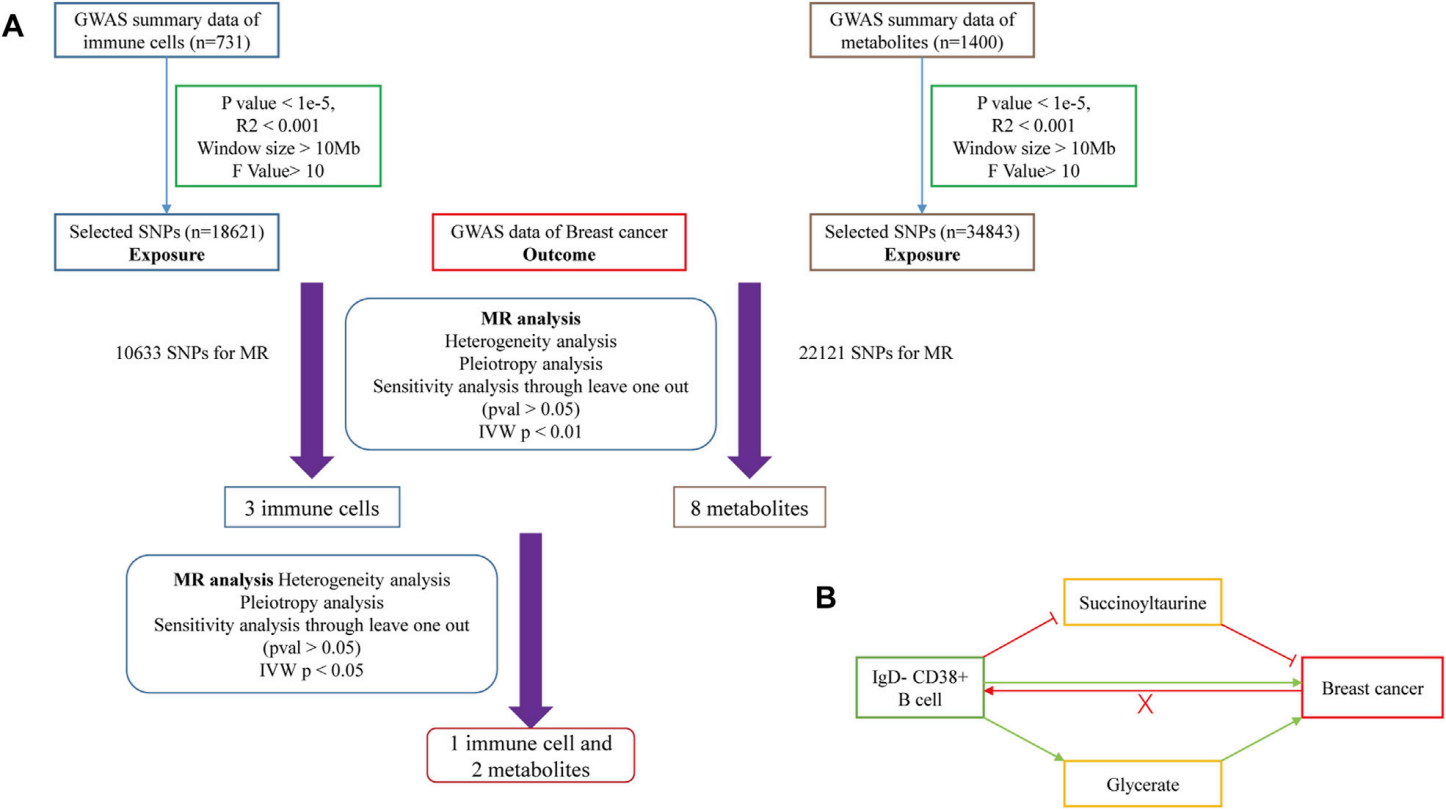
**(A)** Flowchart of Mendelian Randomization analysis conducted in this study. **(B)** Schematic representation of mediation analysis.

To identify instrumental variables (IVs) significantly influencing the risk of BC, we implemented the following strategies. SNPs were selected based on their correlation with the exposure (immune cells or metabolites) using the following criteria: *p* < 1e-5, distance greater than or equal to 10,000 kbp, and exclusion of SNPs with linkage disequilibrium (*r* ^2^ < 0.001). This process yielded 18,621 SNPs for immune cells and 34,843 SNPs for metabolites (Figure 1A) (Pierce et al., 2011). We found that when we set the threshold of P at 5e-8, we got too few SNPs to support our further research. We set our threshold at 1e-5 with reference to the standards in previous studies (Yu et al., 2021; Guo et al., 2022; Wang and Zhang, 2024).

Furthermore F-test was employed to eliminate weak IVs, with a threshold of 10. SNPs with an F-value less than 10 were excluded (Burgess et al., 2011). The F-value was calculated as follows: F = *R* ^2^(N−2)/(1−*R* ^2^) (where *R* ^2^ is the variance and N is the sample size of exposure data). The formula for calculating the *R* ^2^ value is as follows: *R* ^2^ = 2 × (1−MAF) × MAF × β^2^ (where MAF is the minor allele frequency and β is the effect size on the exposure). By implementing these filtering criteria, we aimed to select highly informative and reliable IVs for our MR analysis.

### 2.3 Mendelian randomization analysis

MR analysis was conducted to assess the causal relationship between immune cells and the risk of BC. For significant findings, we performed reverse MR analysis to address potential reverse causality (Figure 1B). In the exposure-outcome analysis, we used MR with at least two SNPs as IVs. Five MR methods were employed to estimate the causal relationship: MR Egger, Weighted Median, inverse-variance weighted (IVW), Simple Mode, and Weighted Mode. IVW is widely recognized for its robustness in causal inference and was selected as the primary method for estimating the causal effect (Bowden et al., 2017).

Simultaneously, Cochran’s Q test was used to detect heterogeneity of MR results, with *p* > 0.05 indicating the absence of heterogeneity (Hemani et al., 2018). Horizontal pleiotropy was assessed using the MR Egger intercept test, and a *p* > 0.05 indicated the absence of horizontal pleiotropy (Shu et al., 2022). Sensitivity analysis was performed through the “leave-one-out” method to evaluate whether the causal relationship between exposures and outcomes was influenced by any single SNP (Jin et al., 2023). All the aforementioned MR analysis processes were conducted using the “TwoSampleMR” and “gwasglue” packages in R version 4.3.2.

### 2.4 Analysis of mediating effect

To investigate potential mediating effects of metabolites in the relationship between immune cells and risk of BC, we conducted a metabolite-mediated mediation analysis within the MR framework. This analysis allowed us to estimate the mediating effect of metabolites and evaluate the hypothesis of indirect causation. We utilized the aggregate correlation statistics of immune cells, metabolites, and the risk of breast cancer to estimate and assess the mediation effect (Figure 1B). The methods employed were based on Huang et al. (Chen et al., 2022). Then, we analyzed the causal relationship between the 1,400 metabolites’ levels and the risk of BC. This analysis identified eight metabolites with significant causal relationships with the risk of BC. We Further investigated the causal relationships between these three immune cells and eight metabolites identified in the previous step. This analysis revealed significant causal relationships between one immune cell and two metabolite levels.

## 3 Results

### 3.1 Study design

The MR analysis workflow is depicted in Figure 1A. We began by screening the GWAS statistics of immune cells using SNPs. Next we conducted an MR analysis to assess the relationship between immune cells and the risk of BC, identifying three immune cells with a causal effect on the risk of BC. Subsequently, directional MR analysis was employed, revealing that BC has no significant causal effect on the levels of these three immune cells. Therefore, we introduced metabolites as potential mediators and conducted MR analysis with BC after SNPs screening, identifying eight metabolites with a causal relationship with the risk of BC. Finally, we utilized the SNPs of these three immune cells as exposure factors and the levels of the eight metabolites as outcome factors for MR analysis. Our findings indicated that one immune cell could collectively increase the risk of BC by elevating the level of one metabolite and reducing the level of another metabolite (Figure 1B).

### 3.2 Selection of instrumental variables

Following established quality control criteria, SNPs associated with immune cells were selected as instrumental variables (IVs) (18,621 SNPs for 731 immune cells, details in Supplementary Table S3). Similarly, 34,843 SNPs associated with 1,400 metabolites were chosen as IVs (details in Supplementary Table S4). The F-test results for these SNPs exceeded the threshold of 10, indicating their strong representation of immune cells and metabolites in the MR analysis. This suggested that the selected IVs were informative and dependable for estimating causal effects.

### 3.3 Causal estimates of the genetic susceptibility to immune cells and breast cancer

**FIGURE 2:**
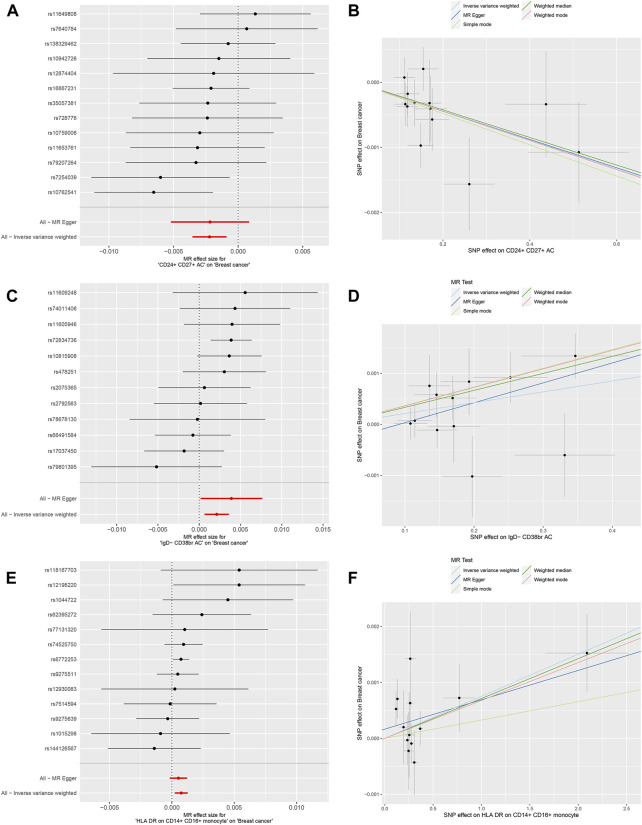
Mendelian randomization analyses for immune-cell traits and risk of breast cancer. **(A,B)** CD24^+^ CD27^+^ B cells. **(C,D)** IgD- CD38^+^ B cells. **(E,F)** CD14^+^ CD16^+^ monocytes.

We conducted MR analysis on 731 immune cells associated with BC. Out of the 731 causal pairs examined, three of these demonstrated statistically significant casual relationships (*p* < 0.01). The IVW method indicated that genetically predicted CD24^+^ CD27^+^ B cells were associated with a decreased risk of BC (OR = 0.9978, 95% CI: 0.996–0.999, *p* = 0.001, Figures 2A, B). As shown in Figures 2C, D, the IVW method revealed that genetically predicted IgD- CD38^+^ B cells were linked to an increased risk of BC (OR = 1.002, 95% CI: 1.001–1.004, *p* = 0.005). Additionally, in Figures 2E, F, the IVW method demonstrated that genetically predicted CD14^+^ CD16^+^ monocytes were also associated with an increased risk of BC (OR = 1.000, 95% CI: 1.000–1.001, *p* = 0.005). Subsequently, three sets of statistically significant samples were utilized to perform a reverse MR study, mitigating the impact of reverse causation.

Furthermore, MR-Egger regression and weighted median analyses supported the consistency of these casual relationships. Reverse MR analysis did not indicate reverse causality (*p* > 0.05). In the Cochran’s Q test, *p*-values were all greater than 0.05 in all 3 MR analyses (Table 2). This heterogeneity among IVs justified the use of the random effect model in these cases. It is worth noting that the intercept of the MR-Egger regression did not deviate from 0, indicating that there was no evidence of pleiotropic IVs level related to immune cells and risk of BC (*p* > 0.05, Table 2). Additionally, the analysis of Leave-one-out analysis confirmed that no single IV significantly influenced causal inferences (Supplementary Figure S1). In summary, These robustness and sensitivity analyses support the reliability of the observed causal relationships between the three immune cell traits and risk of BC.

**TABLE 2:** MR analysis results on the association between immune cells and risk of breast cancer.

Phenotypes	Number of SNPs	MR analysis through IVW				Pleiotropy test			Heterogeneity test			revPvale
		b	se	pval	or	egger_intercept	se	pval	Q	Q_df	Q_pval	
CD24^+^ CD27^+^ AC	13	−0.00221463164848281	0.000673890150989756	0.0010149869010471	0.997787818838877	−7.05641577632687E-06	0.000258431973135011	0.978705754878458	10.0268920907742	12	0.613601326814706	0.940831773832894
IgD- CD38br AC	12	0.00212352089303473	0.000754590897819587	0.00489086363834303	1.00212577716032	−0.000357185530457107	0.000352007985969734	0.334166318847955	12.3930652278852	11	0.334834325193655	0.444807836094989
HLA DR on CD14^+^ CD16^+^ monocyte	13	0.000751625814137905	0.000266305681722602	0.00476630846848242	1.0007519083556	0.000164797364869161	0.00017222399833562	0.359186770389337	10.4782124695095	12	0.574083279519848	0.751195844583616

### 3.4 Identification of metabolites with mediating effects

**FIGURE 3:**
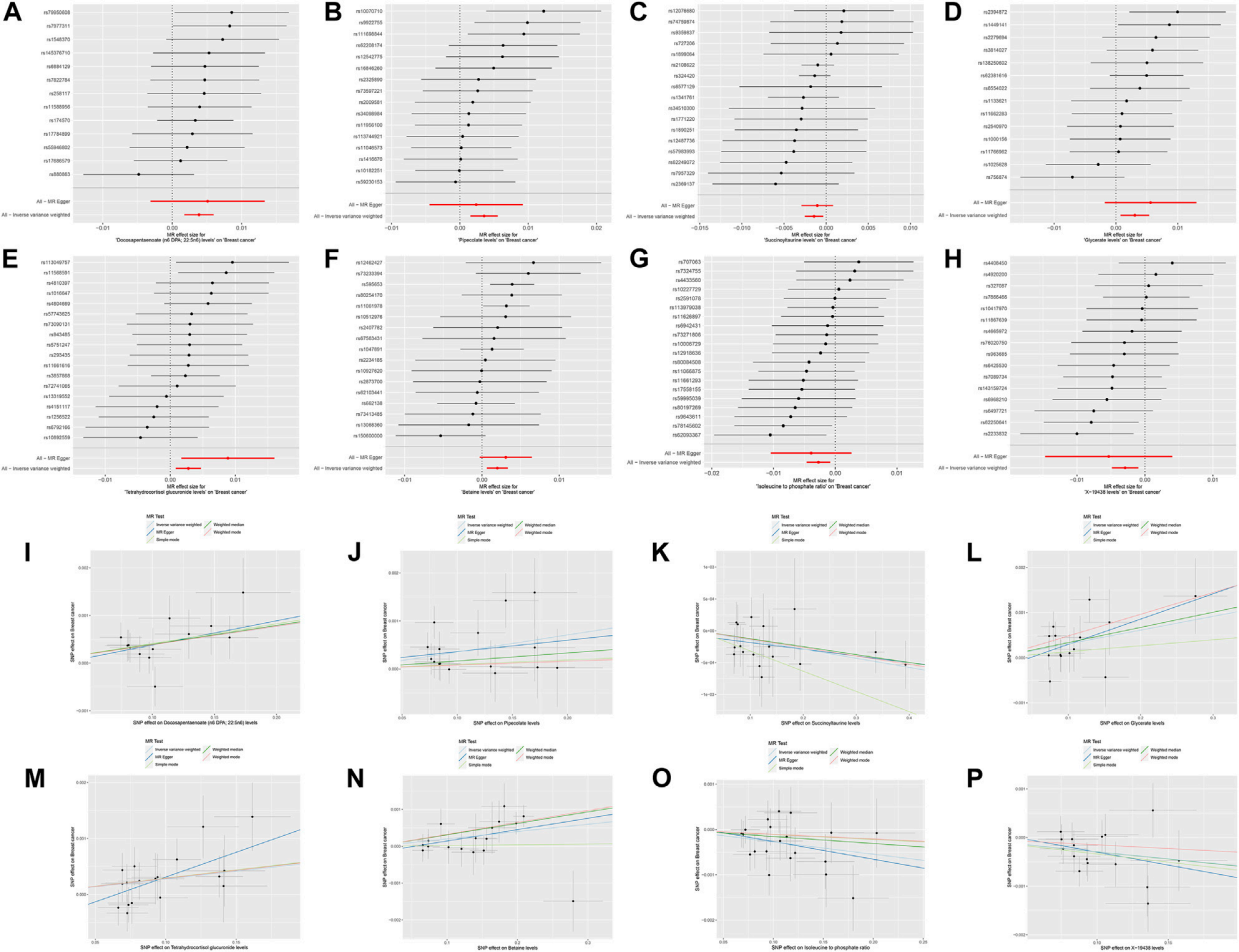
Mendelian randomization analyses for metabolite traits and risk of breast cancer. **(A,I)** Docosapentaenoate (n6 DPA; 22:5n6). **(B,J)** Pipecolate. **(C,K)** Succinoyltaurine. **(D,L)** Glycerate. **(E,M)** Tetrahydrocortisol glucuronide. **(F,N)** Betaine. **(G,O)** Isoleucine to phosphate ratio. **(H,P)** X-19438.

**FIGURE 4:**
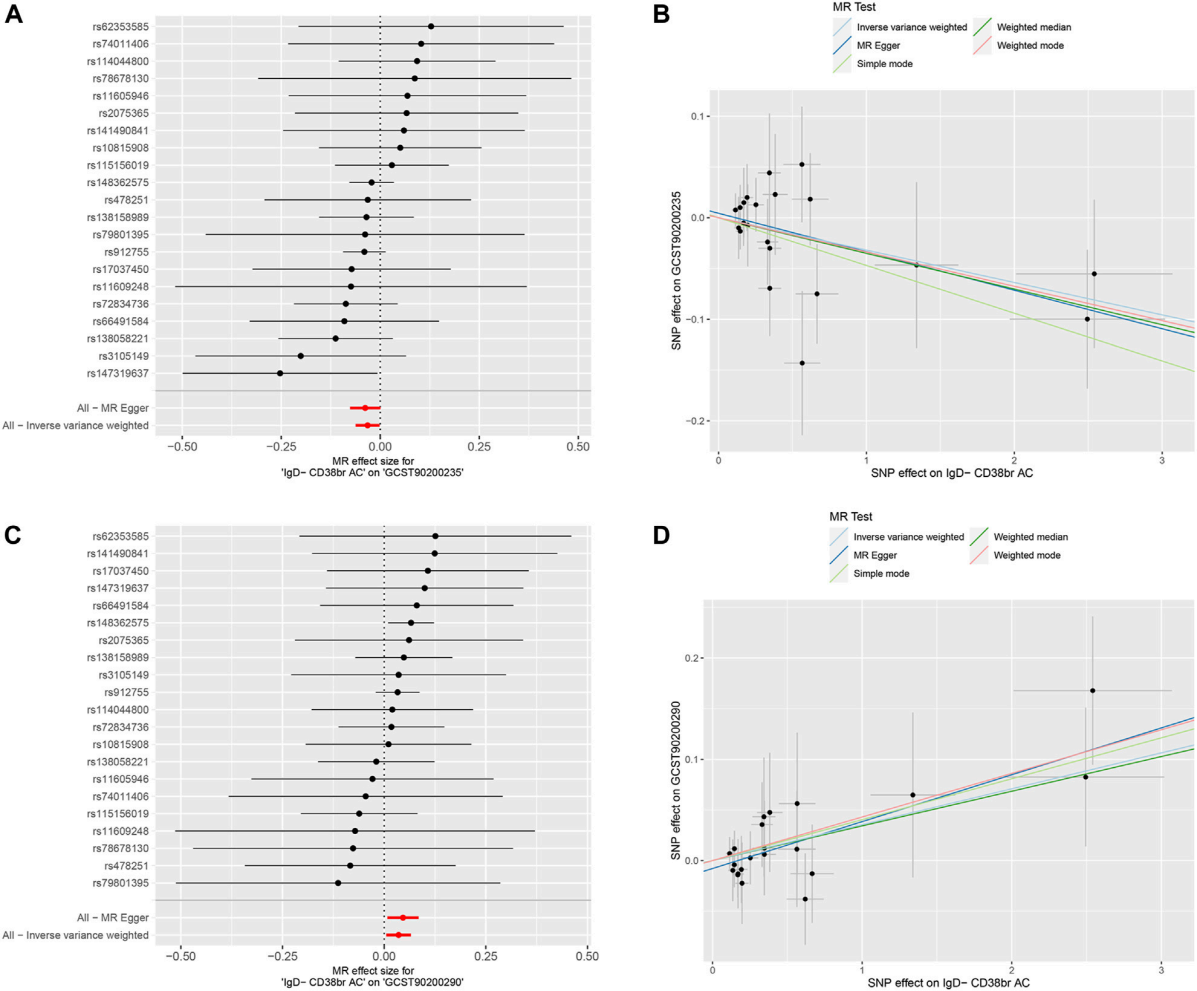
Mendelian randomization analyses for immune-cell traits and metabolite levels. **(A,B)** IgD- CD38^+^ B cells to Succinoyltaurine levels. **(C,D)** IgD- CD38^+^ B cells to Glycerate levels.

Following the establishment of a unidirectional causal relationship between the three immune cells and the risk of BC, we sought to identify metabolites with mediating effects. Initially, we performed MR analysis to screen metabolites exhibiting a significant causal relationship with the risk of BC. Ultimately, eight metabolites were identified based on the following MR screening criteria: 1) IVW analysis method, *p* < 0.01; 2) Cochrane Q test, *p* > 0.05; 3) pleiotropic test and sensitivity analysis, all meeting the requirements of *p* > 0.05 (Figure 3; Supplementary Figure S2). Following this, we investigated the casual effects of the three immune cells on the levels of these eight metabolites. Notably, IgD- CD38^+^ B cells exhibited causal relationships with Succinoyltaurine and Glycerate (*p* < 0.05 in IVW method, Figure 4). Importantly, these SNPs also satisfied the criteria for heterogeneity, pleiotropic tests, and sensitivity analysis (*p* > 0.05, Supplementary Figure S3).

**FIGURE 5:**
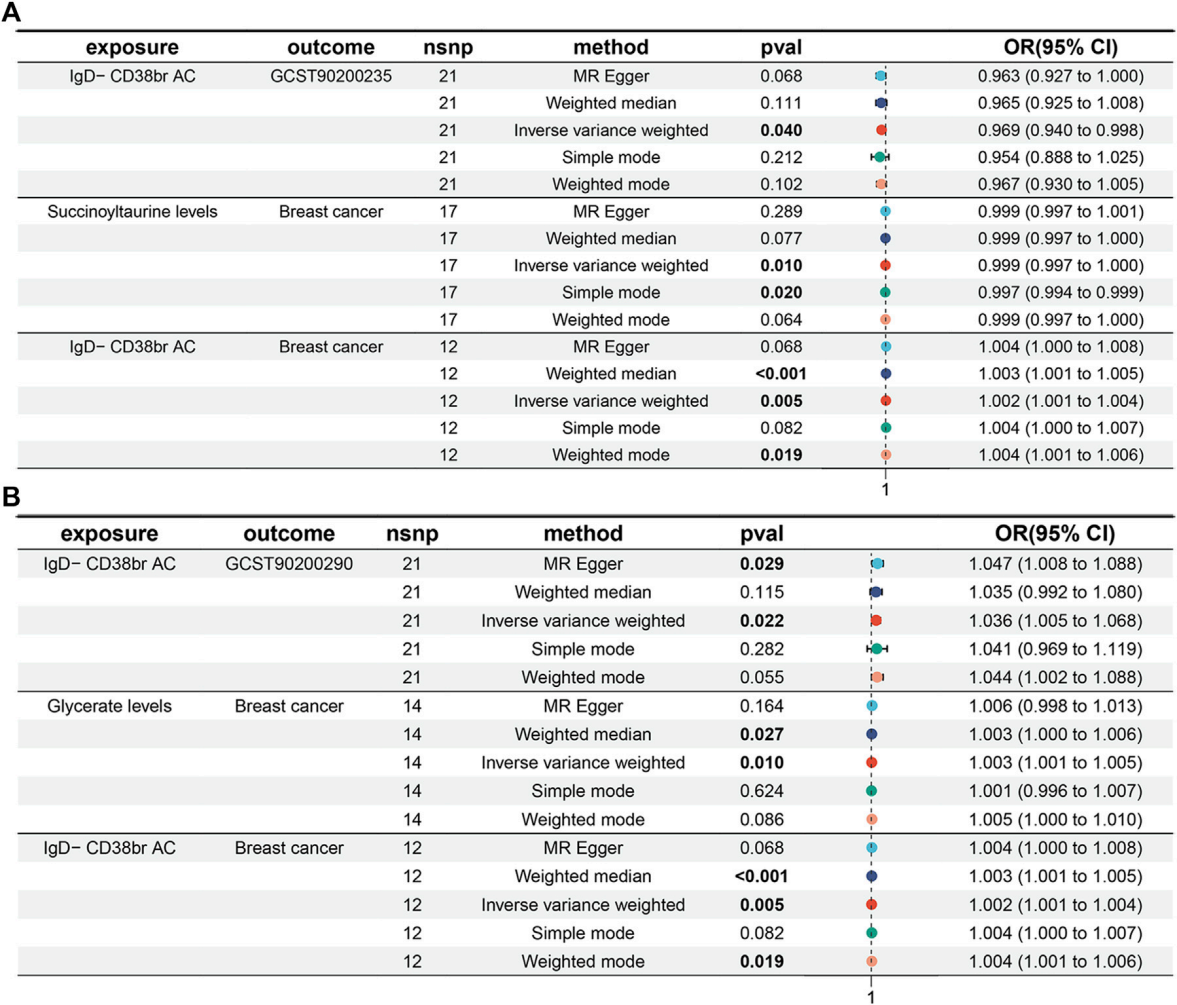
Mediation analyses for the association between IgD- CD38^+^ B cells and risk of breast cancer. **(A)** Mediator: Succinoyltaurine. **(B)** Mediator: Glycerate.

Moreover, a positive causal relationship was identified between IgD- CD38^+^ B cells and Glycerate levels, with the latter also exhibiting a positive causal relationship with the risk of BC (*p* < 0.05 in IVW method, Figure 5). In contrast, IgD- CD38^+^ B cells displayed a negative causal relationship with Succinoyltaurine levels, and the latter also demonstrated a negative causal relationship with the risk of BC. These findings suggest that that IgD- CD38^+^ B cells increased the risk of BC by elevating Glycerate levels and reducing Succinoyltaurine levels (Figure 5).

Gene Otology enrichment analysis and Protein-Protein Interactions (PPI) map of the genes corresponding to SNPs in mediating effect were also worth mentioning (Supplementary Figure S4) through Metascape (Zhou et al., 2019).

## 4 Discussion

With the increasing focus on tumor immunity, researchers are paying growing attention to the role of the Tumor Microenvironment (TME) in tumorigenesis. The alterations in TME extend beyond changes in infiltrated immune cells to include variations in various metabolite levels resulting from the metabolic reprogramming of cells within the TME. Metabolic reprogramming occurs not only in tumor cells but also in stromal cells, including immune cells, within primary tumor tissues. This metabolic reprogramming leads to a TME characterized by increased acidity, nutrient deficiencies, and hypoxia. These conditions not only exacerbate metabolic reprogramming in tumor cells and immune cells but also contribute to the creation of an immunosuppressive microenvironment. This, in turn, regulates immune cells differentiation of and promotes tumor progression (Zheng et al., 2009; Lian et al., 2022). Consequently, further exploration is warranted to better understand the role of metabolic reprogramming in immune cells during the processes of tumorigenesis and tumor progression.

MR relies on three key assumptions. Genetic variants must 1) strongly associate with the exposure factor; 2) not associate with any confounding factors; 3) influence the outcome only through the exposure factor and not through any direct causal pathway. If the assumption 1) is not met, MR analysis cannot be performed; If the assumptions 2) or/and 3) are not satisfied, it may lead to false positive results (Larsson et al., 2023). In this research, we employed a similar approach to the previous studies on SNPs of immune cells and metabolites to obtain IVs for MR analysis and ensure the validity of these three assumptions (Figure 1) (Wang et al., 2024; Wang and Zhang, 2024). In this investigation, we initially identified a causal relationship between IgD- CD38^+^ B cells and an increased risk of BC, whereas no significant causal link was observed between the risk of BC and IgD- CD38^+^ B cells. Subsequently, through an exploration of the mediating effects of metabolites, we uncovered a negative causal association between IgD- CD38^+^ B cells and Succinoyltaurine levels. Simultaneously, there was a negative causal relationship between Succinoyltaurine levels and the risk of BC. Conversely, a positive causal relationship was noted between the levels of IgD- CD38^+^ B cells and Glycerate, and likewise, a positive causal link existed between Glycerate levels and the risk of BC. In summary, we inferred that IgD- CD38^+^ B cells could contribute to an increased risk of BC through both positive and negative mediating effects involving Glycerate and Succinoyltaurine.

BC is infiltrated by various immune cells, typically found in the extracellular matrix or in direct contact with cancer cells (Salgado et al., 2015). Among these immune cells, adaptive immunity mediated by lymphocytes plays a crucial role in eliciting an effective anti-tumor response (Galon et al., 2013). While T lymphocytes primarily fulfill an anti-tumor immune function, recent research has highlighted the increasingly recognized role of infiltrating B lymphocytes in BC, with CD20^+^ B lymphocytes serving as a notable representative (Hussein and Hassan, 2006). CD20^+^ B lymphocytes contribute to an anti-tumor response by generating antibodies against BC antigens, releasing immunogenic cytokines and chemokines, and presenting antigens to T cells (Brown et al., 2014; Sarvaria et al., 2017). Nonetheless, a subset of B cells exists that fosters tumor growth by promoting inflammation and immunosuppression. This is achieved through the secretion of anti-inflammatory and angiogenic mediators, as well as interactions involving immune complexes and complement activation (Yuen et al., 2016). The IgD- CD38^+^ B cells identified in our study belong to a subtype of inhibitory B cells that can elevate the risk of BC through mediating effects (with metabolites as mediators). It has been observed that the heightened infiltration levels of IgD- CD38^+^ B cells were associated with increased inflammation (Wang et al., 2022). In regulatory B cells (Bregs) with IL10+ IgD- CD38^+^, PPARδ was significantly upregulated. However, the immunosuppressive function of IL10+ IgD- CD38^+^ Bregs could be blocked, and cancer immunotherapy enhanced, by using PPARδ inhibitors (Chen et al., 2023b). Overall, research on IgD- CD38^+^ B cells is still limited at present, and further in-depth study is required to fully understand its function and role in tumorigenesis and tumor immunity.

Single nucleotide polymorphisms (SNPs) play a pivotal role in BC. Existing research indicated a close association between the polymorphism at SNPs and the risk of BC (Jupe et al., 2001; Arancibia et al., 2021). These variations may influence gene expression, thereby impacting crucial biological processes such as cell proliferation, differentiation, and apoptosis (Morales-Pison et al., 2021). SNPs variations could lead to alterations in protein structure or function, consequently disrupting the normal regulation of cellular signaling pathways and promoting the occurrence and progression of BC (Noguchi et al., 2009). Moreover, SNPs may influence the response of patients with BC to treatment. Some SNPs could affect the activity of drug-metabolizing enzymes, thus impacting drug concentrations and efficacy *in vivo*, ultimately affecting treatment effects and prognosis for patients with BC (Noguchi et al., 2009; Duong et al., 2023; Oliva et al., 2023). These genes as mentioned, which could be influenced by SNPs and had an impact on the pathogenesis and treatment of BC, include non-coding genes and genes encoding protein, such as those in our research. In summary, SNPs play a significant role in the occurrence, development, and treatment of BC. Further in-depth research into the mechanisms of action of these SNPs will contribute to a better understanding of the pathophysiological processes of BC, providing a theoretical basis and clinical guidance for personalized treatment approaches.

Although we endeavored to enhance the objectivity and credibility of our research during the design stage, we acknowledge certain limitations and shortcomings. Despite utilizing genetic statistics from the open GWAS database, it is important to note that these data might not encompass all ethnic groups and populations, as our focus was primarily on the European population. Since the current GWAS datasets of immune cells and metabolites were only from European population, to ensure the same genetic background, we chose breast cancer GWAS data from European population. We also expect more GWAS datasets of other people (such as Asians, Africans, etc.) to verify our results in the future. MR analysis provides robust evidence of causality, but it relies on pivotal assumptions, including the absence of genetic confounding factors. Any violation of these assumptions may impact the accuracy of the results. Moreover, our research assumes a linear relationship between exposures and outcomes. However, we cannot assess potential nonlinear relationships. Although our analysis identified Succinoyltaurine and Glycerate as mediating factors, the regulation and influence of metabolic levels were intricate, implying the possible existence of other unconsidered mediating factors. While our study revealed a causal relationship between IgD- CD38^+^ B cells and the risk of BC, these results should be cautiously interpreted in the clinical context. Further research is needed to validate our findings and determine the clinical implications. Lastly, our study aims to generate new hypotheses rather than definitive conclusions, and there is a risk of false positives if multiple tests are not corrected. Therefore, our results should be viewed as preliminary and require further validation in future research.

## 5 Conclusion

This MR study provides novel genetic evidence supporting a causal relationship between IgD- CD38^+^ B cells and the risk of BC. Through mediation analysis, we identified that IgD- CD38^+^ B cells contribute to an increased risk of BC through both positive and negative mediation effects involving Glycerate and Succinoyltaurine. Overall, these findings suggest the potential for clinicians to assess the risk of BC in patients by measuring the levels of blood IgD- CD38^+^ B cells. Nevertheless, further clinical research and experiments are warranted to validate these findings in the future.

## Data Availability

The GWAS data of breast cancer presented in the study are deposited in the GWAS IEU, accessing ID: ukb-b-16890 (https://gwas.mrcieu.ac.uk/). The GWAS data of immune cells presented in the study are deposited in the GWAS IEU, accessing ID: ebi-a-GCST0001391 to ebi-a-GCST0002121 (https://gwas.mrcieu.ac.uk/). The GWAS data of metabolites presented in the study are deposited in the GWAS Catalog, accessing ID: GCST90199621 to GCST90201020 (https://www.ebi.ac.uk/gwas/).

## Supplementary material

SUPPLEMENTARY FIGURE S1 Leave-one-SNP-out sensitivity analyses for the Mendelian randomization analyses of immune-cell traits and risk of breast cancer. **(A)** CD24+ CD27+ B cells. **(B)** IgD- CD38+ B cells. **(C)** CD14+ CD16+ monocytes.

SUPPLEMENTARY FIGURE S2 Leave-one-SNP-out sensitivity analyses for the Mendelian randomization analyses of metabolite traits and risk of breast cancer. **(A)** Docosapentaenoate (n6 DPA; 22:5n6). **(B)** Pipecolate. **(C)** Succinoyltaurine. **(D)** Glycerate. **(E)** Tetrahydrocortisol glucuronide. **(F)** Betaine. **(G)** Isoleucine to phosphate ratio. **(H)** X-19438.

SUPPLEMENTARY FIGURE S3 Leave-one-SNP-out sensitivity analyses for the Mendelian randomization analyses of immune-cell traits and metabolite levels. **(A)** IgD- CD38+ B cells to Succinoyltaurine levels. **(B)** IgD- CD38+ B cells to Glycerate levels.

SUPPLEMENTARY FIGURE S4 GO enrichment analysis and Protein-Protein Interactions (PPI) of the gene of Mendelian randomized SNPs of breast cancer by immune cells **(A)** and metabolites **(B)**.

SUPPLEMENTARY TABLE S1 731 immune cells included in the study.

SUPPLEMENTARY TABLE S2 1400 metabolites included in the study.

SUPPLEMENTARY TABLE S3 SNPs associated with immune cells chosen as IVs.

SUPPLEMENTARY TABLE S4 SNPs associated with metabolites selected as IVs.
