# Supplementary figures and images for: Causal effects and metabolites mediators between immune cell and risk of breast cancer: a Mendelian randomization study
Source: Front Genet. 2024 May 17;15:1380249. doi: 10.3389/fgene.2024.1380249 (PMC11140059; doi:10.3389/fgene.2024.1380249)

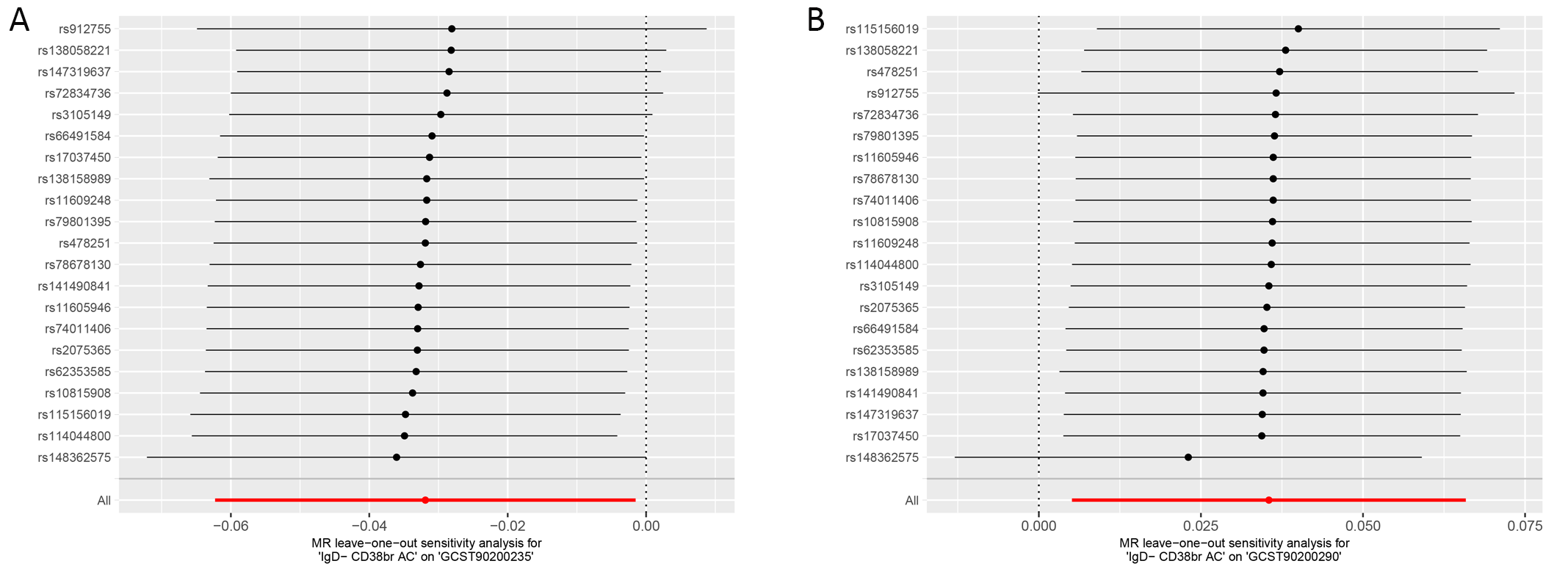

Supplement: Supplementary file 1 [file Image3.TIF]

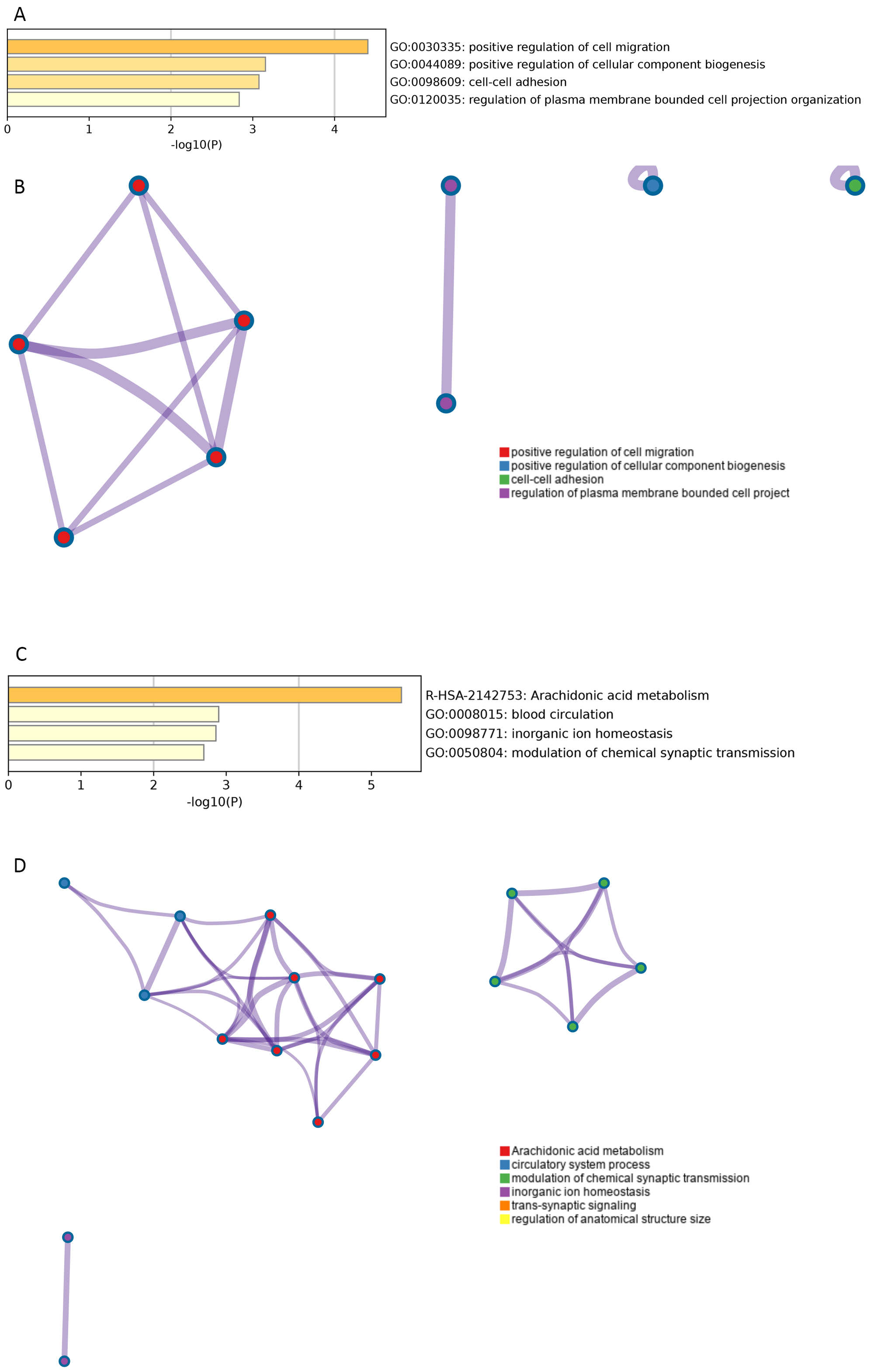

Supplement: Supplementary file 2 [file Image4.TIF]

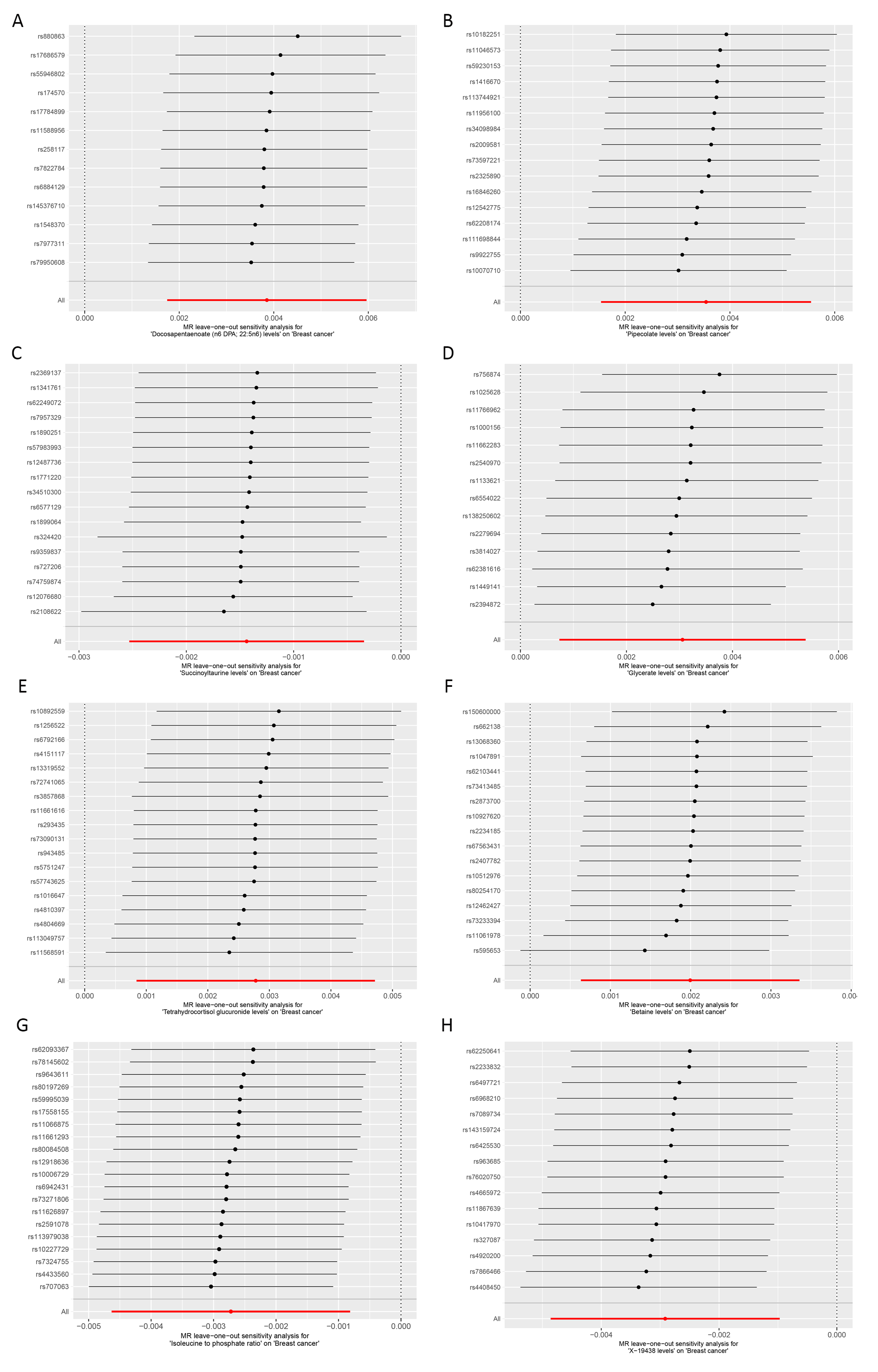

Supplement: Supplementary file 3 [file Image2.TIF]

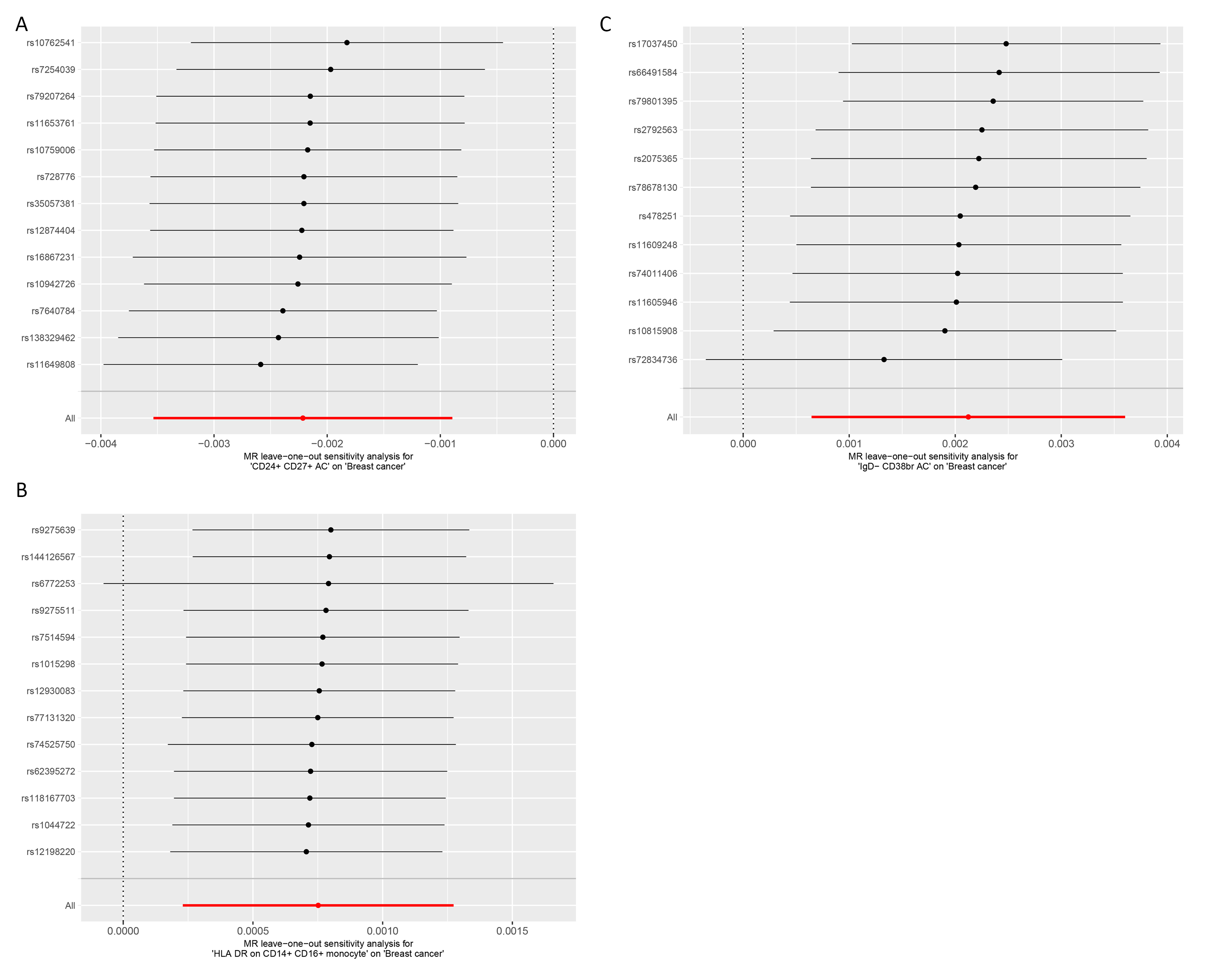

Supplement: Supplementary file 4 [file Image1.TIF]
